# Small GTPase RHOE/RND3, a new critical regulator of NF‐κB signalling in glioblastoma multiforme?

**DOI:** 10.1111/cpr.12665

**Published:** 2019-07-22

**Authors:** Qian Sun, Huimin Dong, Yuntao Li, Fan'en Yuan, Yang Xu, Shanping Mao, Xiaoxing Xiong, Qianxue Chen, Baohui Liu

**Affiliations:** ^1^ Department of Neurosurgery Renmin Hospital of Wuhan University Wuhan China; ^2^ Central Laboratory Renmin Hospital of Wuhan University Wuhan China; ^3^ Department of Neurology Renmin Hospital of Wuhan University Wuhan China; ^4^ Department of Cell Biology, Hubei Key Laboratory of Cell Homeostasis, College of Life Sciences Wuhan University Wuhan China; ^5^ Department of Neurosurgery Huzhou Central Hospital Huzhou China

**Keywords:** apoptosis, glioblastoma multiform, NF‐κB signalling, p65, RND3

## Abstract

**Objectives:**

Abnormal activation of NF‐κB signalling is a major mechanism of apoptosis resistance in glioblastoma multiforme (GBM). Therefore, better understanding of the regulation of NF‐κB signalling has a significant impact for GBM therapy. Here, we uncovered a critical role of the small GTPase RND3 in regulating the p65 subunit of NF‐κB and NF‐κB signalling in GBM.

**Materials and methods:**

Human GBM samples, GBM cells and a human orthotopic GBM‐xenografted animal model were used. The mechanisms of RND3 in regulation of NF‐κB signalling and GBM cell apoptosis were examined by luciferase assay, quantitative PCR, immunostaining, immunoblotting, immunofluorescence, coimmunoprecipitation, TUNEL staining, JC‐1 analysis and flow cytometry.

**Results:**

Overexpression of RND3 led to reduced p65 activity in GBM‐cultured cells and a GBM animal model, indicating that the NF‐κB pathway is negatively regulated by RND3 in GBM. Mechanistically, we found that RND3 bound p65 and promoted p65 ubiquitination, leading to decreased p65 protein levels. Furthermore, RND3 enhanced cleaved caspase 3 levels and promoted apoptosis in GBM cells, and RND3 expression was positively correlated with cleaved caspase 3 and IL‐8 in human GBM samples. The effect of RND3 on promoting apoptosis disappeared when p65 ubiquitination was blocked by protease inhibitor carfilzomib or upon co‐expression of ectopic p65.

**Conclusions:**

RND3 binds p65 protein and promotes its ubiquitination, resulting in reduced p65 protein expression and inhibition of NF‐κB signalling to induce GBM cell apoptosis.

## 1 INTRODUCTION

Glioblastoma multiforme (GBM) is one of the most malignant tumours in the adult central nervous system. GBM tumours show characteristics of malignant cell proliferation, apoptosis inhibition and uncontrollable invasion. GBM patients who undergo surgery followed by post‐operative radiotherapy and chemotherapy still have an average survival time of <2 years.^1^ A contributing factor to the lack of effective treatment strategies and poor prognosis of GBM patients is our limited understanding of the mechanisms of GBM occurrence and development. Previous studies have shown that NF‐κB signalling plays a major role in promoting tumour pathobiology and the response to therapy in GBM.^2^

As a nuclear transcription factor, NF‐κB is expressed in a wide variety of cell types and binds to specific promoter sequences of various genes to facilitate gene transcription and protein expression. In various types of tumours, NF‐κB is the centre of intracellular signal transduction and involved in cell proliferation, apoptosis and many other physiological and pathological regulation processes.^3^

NF‐κB is a family of related multi‐subunit transcription factors, including p65 (RelA), RelB, c‐Rel, p50/p105 (NF‐κB1) and p52/p100 (NF‐κB2).^3^ In the recent study, the activity of the NF‐κB signalling pathway in tumours was likely to be activated by the p65/p50 dimer. Through its binding to the inhibitor IκB‐α, the p65/p50 dimer is maintained an inactive state in the cytoplasm. When IκB‐α is degraded via ubiquitination, the p65/p50 dimer enters the nucleus and activates downstream genes, such as interleukin (IL)‐8, monocyte chemoattractant protein (MCP)‐1 and interleukin (IL)‐6 genes.^4^

Although NF‐κB was originally characterized within the context of the immune system, NF‐κB was subsequently identified as a critical factor in GBM occurrence and development,^5^ and therefore as a critical target for GBM therapy. To date, studies examining NF‐κB as a potential therapeutic target have focused on non‐specific agents or on targets such as IκK that affect multiple signalling pathways.^2^ However, although a number of approaches targeting NF‐κB have shown promise in pre‐clinical GBM models, the strategies that have been tested in clinical settings have thus far not shown satisfactory advantages over other approaches.^5^ Therefore, strategies that manipulate specific subsets of NF‐κB signalling may be more successful for treating GBM.^2^

In this study, we revealed a new mechanism for regulation of the NF‐κB signalling pathway in GBM, which involves RND3. RND3/RhoE is an important member of the Ras superfamily and an atypical Rho‐GTP that lacks GTP hydrolytic activity.^6^, ^7^ We found that overexpressed RND3 directly bound to p65 and promoted p65 ubiquitination to reduce p65 protein levels. RND3 expression led to inhibition of the NF‐κB signalling pathway and reduced expression of its downstream target genes, *IL‐8* and *B‐cell lymphoma/leukaemia (BCL)‐2*, along with increased apoptosis. In contrast, knockdown of RND3 reduced the binding of RND3 to p65 protein and decreased p65 protein ubiquitination to increase p65 protein expression. This promoted activation of the NF‐κB signalling pathway and overexpression of its downstream target genes *IL‐8* and *BCL‐2*, leading to apoptosis resistance in GBM cells.

## 2 MATERIALS AND METHODS

The study materials can be made available for the purposes of reproducing the results or replicating the procedures. The detailed methods and any associated references are provided in the Appendix S1.

### 2.1 Human GBM samples and control brain tissues

Human GBM samples were obtained from GBM tumour tissues of patients. The samples were collected from the same site of tissue collection for pathological examination. All tumour samples were subjected to pathological examination and related molecular testing (MGMT, 1p19q and IDH1/IDH2), all defined according to the 2016 WHO classification.^8^ Normal brain samples were collected from brain tissues removed from trauma patients during surgical decompression or brain tissues removed from the path followed to reach the GBM tumour by the surgeon during tumour removal in GBM patients. All sample information is shown in the Table S1. Written informed consent was obtained from patients or relatives in the case of patients with impaired consciousness prior to sample collection. This study was approved by the Institutional Ethics Committee of the Faculty of Medicine at Renmin Hospital of Wuhan University [approval number: 2012LKSZ (010) H].

### 2.2 Statistical analysis

Data were expressed as the mean ± standard error of the mean (SEM). Statistical analysis was performed with SigmaPlot version 11.0 and SPSS 13.0. Differences between the mean of two groups were assessed by Student's *t* test, and differences in the mean of multiple groups were assessed by one‐way ANOVA. Correlations of two groups and comparisons of quantitative values of expression were assessed by Pearson's test. A value of *P* < .05 was considered to indicate a statistically significant difference. All experiments were repeated three times.

## 3 RESULTS

### 3.1 RND3 inhibits the NF‐κB pathway both in vitro and in vivo

**Figure 1:**
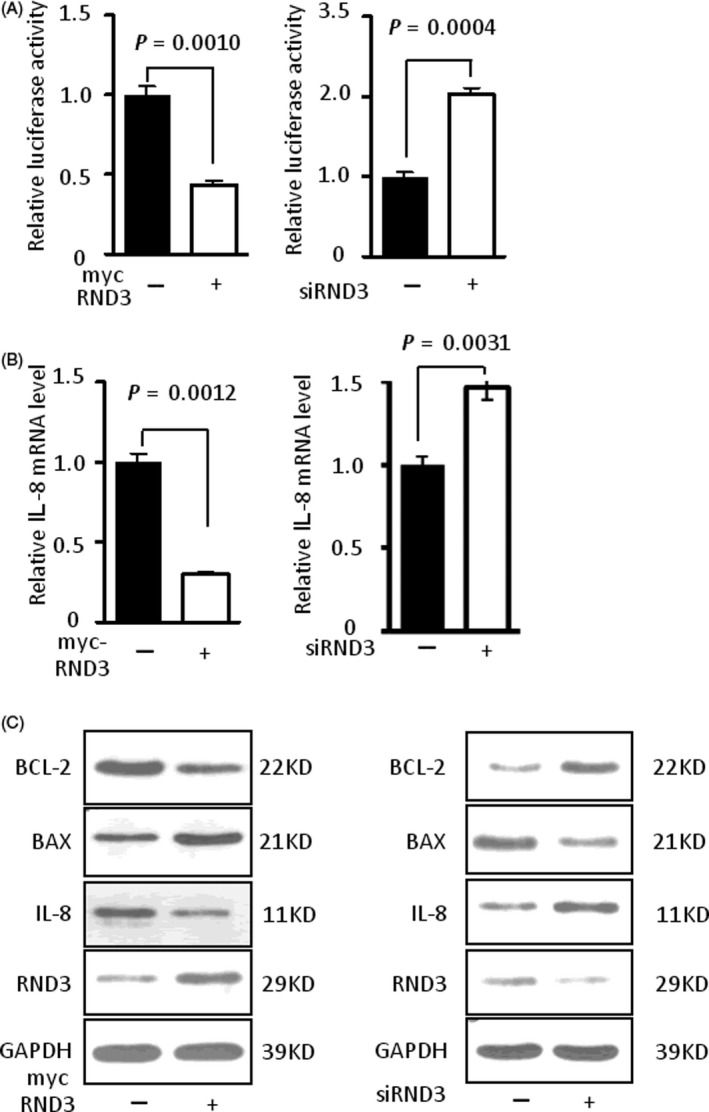
RND3 inhibits NF‐κB signalling. A, Relative luciferase activity of a luciferase reporter driven by a promoter containing p65 binding sites after overexpression or downregulation of RND3 by transfecting an RND3 plasmid (myc‐RND3) or siRNA‐specific targeting RND3 (siRND3) into U87 human GBM cells. B, Relative *IL‐8* mRNA level after overexpression or downregulation of RND3 in U87 cells. C, BAX, BCL‐2 and IL‐8 protein expression levels after overexpression or downregulation of RND3 in U87 cells. myc‐RND3: overexpression of RND3 by transfection of the myc‐RND3 plasmid. myc: vector control plasmid. siRND3: siRNA SMARTpool specific knock down RND3 in U87 cells, siCtrl: vector control siRNA SMARTpool

A plasmid for overexpression of RND3 or SMARTpool to specifically knock down RND3 was transfected into U87 and U251 cells, and immunoblotting confirmed successful overexpression or downregulation of RND3. We used a luciferase reporter assay to examine the activity of NF‐κB and result showed that the luciferase activity decreased significantly (*P* < .05) in the RND3‐overexpressing group compared with the control group, while the opposite effects were observed with RND3 downregulation (Figures 1A and S1A).

IL‐8 is an important target of NF‐κB signalling and its gene expression mostly regulated by NF‐κB.^9^, ^10^ Therefore, we used IL‐8 as a reporter for NF‐κB signalling in vivo and in vitro. Compared with the control group, high expression of RND3 significantly decreased *IL‐8* mRNA expression (*P* < .05), while downregulation of RND3 led to increased *IL‐8* mRNA levels in both U87 and U251 cells (Figures 1B and S1B). These data were supported by immunoblots showing that protein expression of IL‐8 was decreased when RND3 was overexpressed, while reduced expression of RND3 elevated the expression of IL‐8 (Figures 1C and S1C). In addition, BCL‐2 and the BCL‐2‐associated X protein (BAX), apoptotic factors that are also mostly regulated by NF‐κB signalling,^2^ were also examined by immunoblotting and real‐time PCR. The expression of BCL‐2 was decreased and BAX expression was increased when RND3 was overexpressed in both mRNA and protein level in U87 and U251 cells, and reduced levels of RND3 resulted in the opposite effects (Figures 1C, S1C and S4A,B).

**Figure 2:**
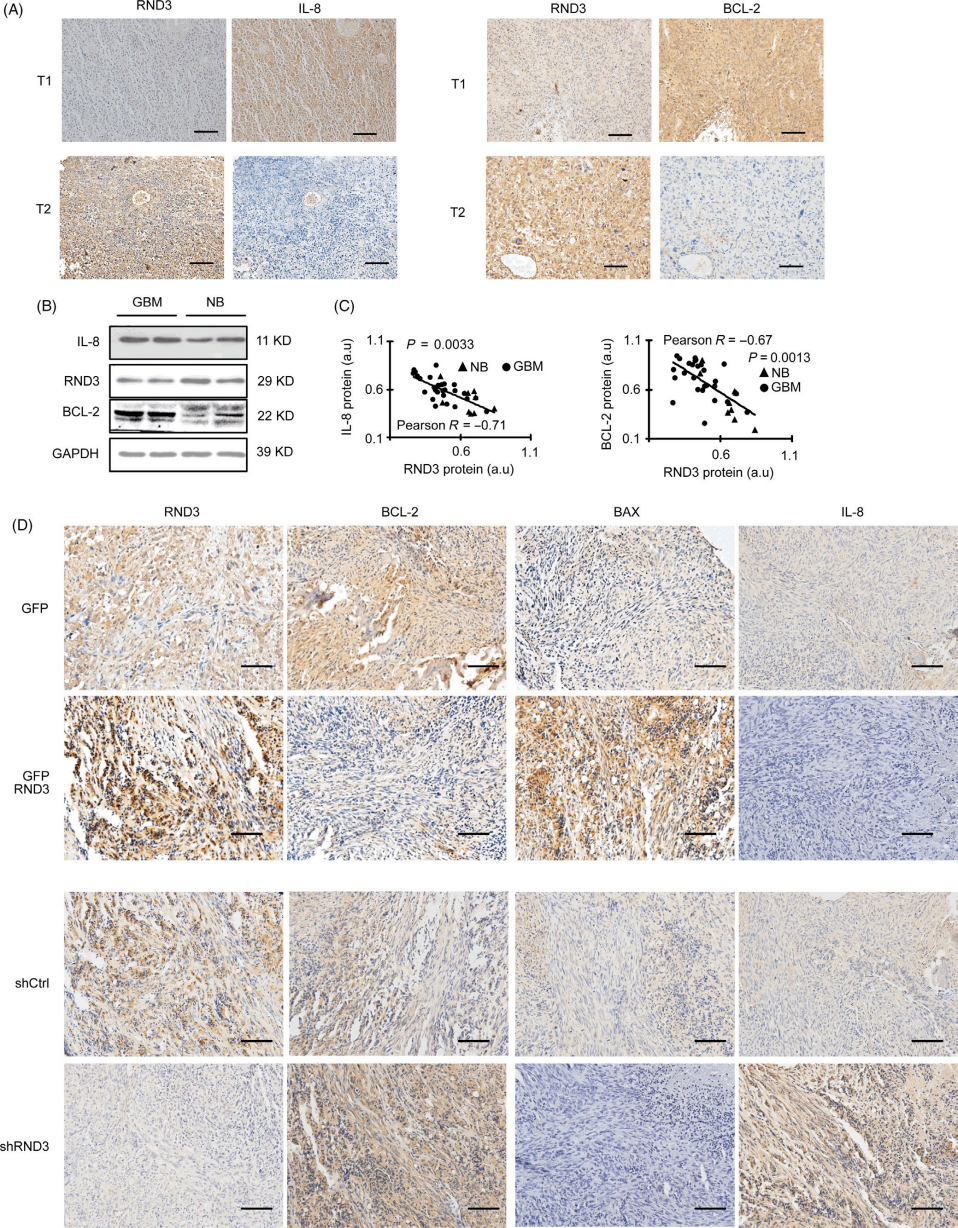
RND3 expression negatively correlates with IL‐8 and BCL‐2 expression in human GBM cells and implanted orthotopic tumours in nude mice. A, Immunohistochemical staining of RND3 and IL‐8 in the same region of human GBM tissues. B, Immunoblotting of RND3, Bcl‐2 and IL‐8 in the same region of human GBM tissues. C, Quantitative analyses of RND3 and Bcl‐2, IL‐8 in 27 GBM tissues and nine normal brain tissues (NB). D, Immunostaining of BCL‐2, IL‐8 and BAX in implanted orthotopic tumours of nude mice in the indicated groups. GFP‐RND3 group: mice were injected with U251 cells stably expressing GFP‐RND3 (n = 12); GFP group: mice were injected with U251 cells stably expressing GFP (n = 12); shRND3 group: mice were injected with U251 cells stably expressing shRNA targeting RND3 (shRND3) (n = 12); shCtrl group: mice were injected with U251 cells stably expressing control shRNA (shCtrl) (n = 12). Scale bar = 100 μm. Triangle represents normal brain tissue. Circles represent human GBM tissue. a.u., arbitrary unit; T1, tumour 1; T2, tumour 2

To further analyse the relationship between RND3 and NF‐κB signalling in GBM, RND3 and IL‐8 expressions were assessed by immunohistochemical analyses in GBM tissues. The results showed that the expression of IL‐8 was increased together with a decrease of RND3 in the same regions of human GBM tissues (Figure 2A). Immunoblot analyses of 27 human GBM and nine human brain specimens showed that RND3 was inversely associated with IL‐8 protein expression (Figure 2B,C).

To further support these data, the positive regulation of RND3 in NF‐κB signalling was also examined in vivo in the human orthotopic GBM‐xenografted animal model. Intracranial implantation of U251 GBM cells with stable overexpression of RND3 or control cells was performed in nude mice, and BCL‐2, IL‐8 and BAX expressions were examined in the mouse model by immunohistochemical and real‐time PCR analyses. The results showed that BCL‐2 and IL‐8 expression was decreased, while BAX expression was increased in the RND3‐overexpressing group, and BCL‐2 and IL‐8 expression was increased, while BAX expression was decreased in the RND3‐downregulated group in both protein and mRNA level (Figures 2D, S2 and S4C,D,E). Collectively, these data indicate that the NF‐κB pathway is negatively regulated by RND3 in GBM.

### 3.2 RND3 decreases p65 protein expression to inhibit NF‐κB signalling

**Figure 3:**
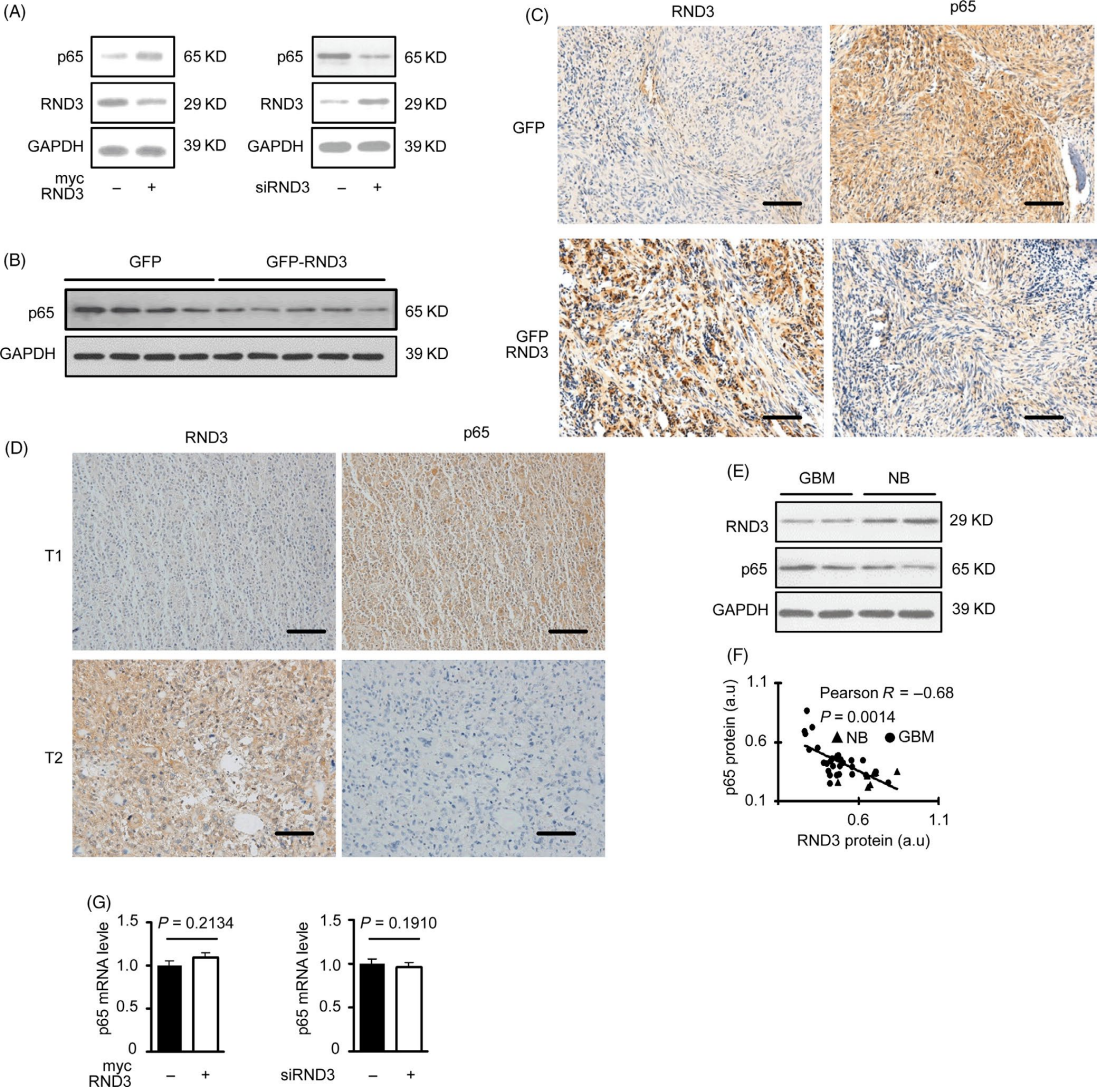
RND3 decreases expression of p65 protein but not mRNA in vivo and in vitro. A, Immunoblotting of p65 and RND3 proteins after up‐ or downregulation of RND3 in GBM cells U87. B, C, Immunostaining (brown) and immunoblot analysis of p65 protein in nude mouse tumours (n = 12 for each group). D, Representative immunostaining (brown) of RND3 and p65 in the same region of human GBM tissues. Blue indicates nuclear staining. E, Immunoblot analysis of RND3 and p65 in human GBM and normal brain tissues. F, Correlation between changes in RND3 and p65 protein levels of human GBM (circles, n = 27) and normal brain (triangle, n = 9) tissues. G, Quantitative PCR analysis of p65 mRNA expression after up‐ or downregulation of RND3 in GBM cells U87. Scale bar: 100 μm. a.u., arbitrary unit; T1, tumour 1; T2, tumour 2

P65 plays a key role in NF‐κB signalling.^3^ To determine the mechanism of RND3 regulating NF‐κB signalling, we examined p65 levels after RND3 overexpression or knockdown in U87 and U251 cells. We found that p65 protein expression level was decreased by RND3 overexpression, while downregulation of RND3 led to increased p65 levels (Figures 3A and S3A), indicating that RND3 decreased p65 protein expression in human GBM cells. We next examined p65 expression by immunostaining and immunoblotting in the human GBM‐xenografted mouse model. The results showed that p65 expression was decreased in the RND3‐overexpressing group compared with the control group (Figures 3B,C and S3B).

We also evaluated the expressions of RND3 and p65 by immunoblotting in human GBM tissues. The results showed that the expression of p65 was increased together with a decrease of RND3 in the same regions of GBM specimens (Figure 3D). Furthermore, immunostaining analyses of 27 human GBM and 9 human brain specimens showed that RND3 was inversely associated with p65 protein expression in 36 human specimens (Figure 3E,F).

Collectively, these data suggested that RND3 may inhibit NF‐κB signalling in human GBM through downregulating p65 protein levels.

### 3.3 RND3 decreases p65 protein expression by increasing ubiquitination of p65 protein

**Figure 4:**
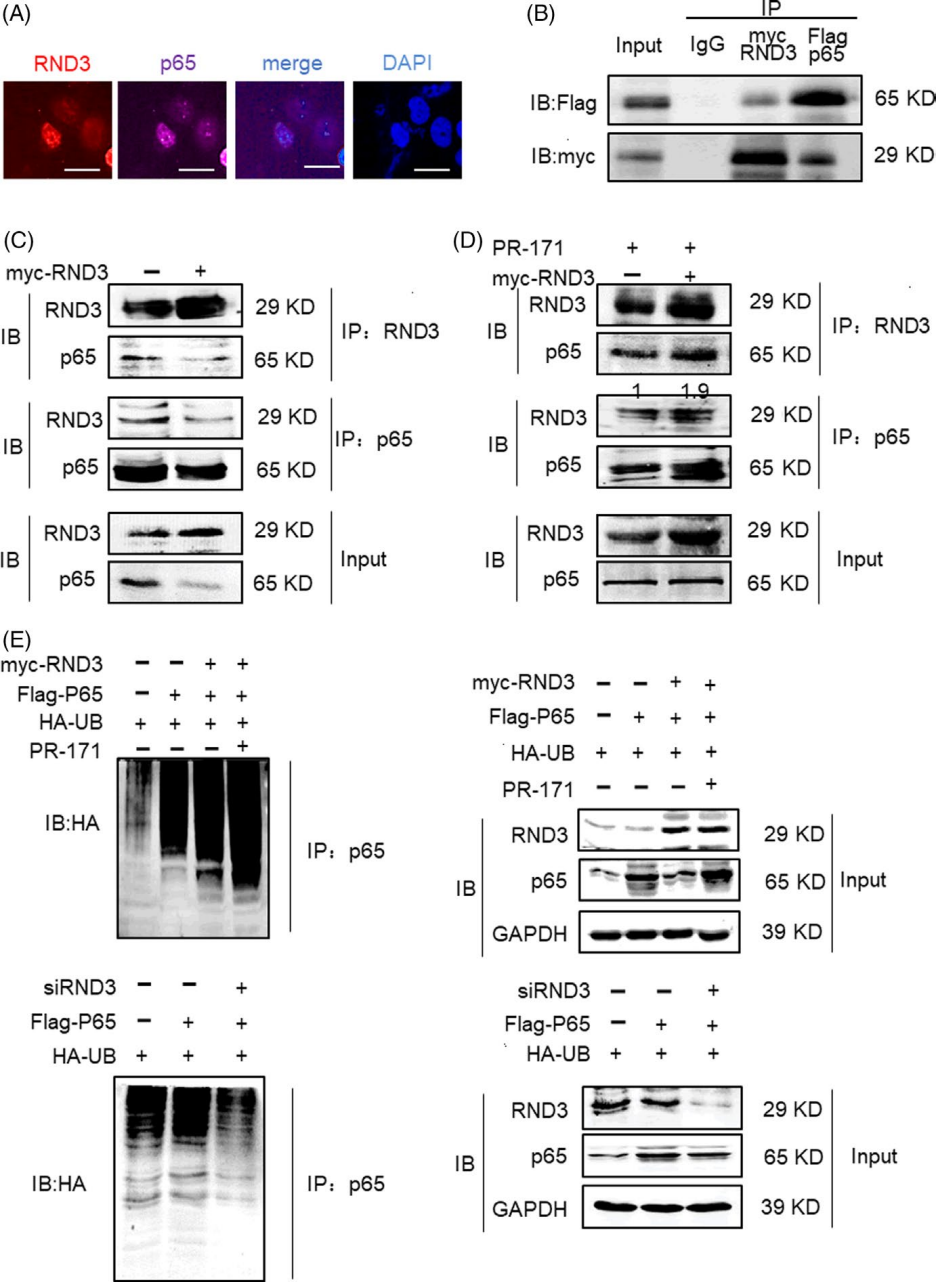
RND3 binds directly to p65 and degrades p65 protein through the ubiquitin‐mediated proteosomal pathway. A, Confocal microscopy of RND3 and p65 localizations in GBM cells (scale bar = 20 µm). B, Coimmunoprecipitation assay in U87 cells co‐transfected with a myc‐RND3 expression construct and Flag‐tagged p65 in U87 cells. C, Coimmunoprecipitation of RND3 and endogenous p65 in myc‐RND3‐transfected U87 cells and controls U87 cells transfected with vector control plasmid (myc). D, Coimmunoprecipitation of RND3 and endogenous p65 in myc‐RND3‐ and control‐transfected U87 cells treated with PR‐171 (20 nm). E, Representative immunoblots of p65 and ubiquitinated p65 in U87 cells when RND3 was up‐ or downregulated and PR‐171 was used to treat U87 cells. IP: specific antibody used to pulled down proteins; IB: specific antibody used to detected protein expression level by Western blot

To explore the mechanism of RND3 decreasing p65 protein expression, we first measured the p65 mRNA expression level after overexpressing or downregulating RND3 in human U87 cells. We found that p65 mRNA expression was not affected by RND3 levels (Figure 3G), indicating that RND3 does not impact p65 mRNA transcription and may instead regulate p65 protein expression post‐transcriptionally. To further evaluate the mechanism of RND3 regulating p65, confocal microscopy was next used to observe the localizations of RND3 and p65 in GBM cells. The results showed that the subcellular localizations of RND3 and p65 mostly overlapped (Figure 4A).

Furthermore, coimmunoprecipitation confirmed an interaction between ectopically expressed Flag‐p65 and myc‐RND3 protein (Figure 4B). Expression of RND3 and p65 was not detected in immunoprecipitations using non‐specific antibodies. We also performed coimmunoprecipitation mutual pull‐down assays in both myc and myc‐RND3 groups. The results demonstrated a physical interaction between RND3 and p65 in GBM cells at the endogenous level in myc‐RND3(−) group (Figure 4C). In cells overexpressing RND3, the levels of p65 in lysates and co‐immunoprecipitated with RND3 were decreased. However, when both RND3‐overexpressing and control cells were treated with carfilzomib (PR‐171), a proteasome inhibitor,^11^ the levels of p65 in whole‐cell lysates and co‐immunoprecipitated with RND3 increased (Figure 4D).

P65 protein expression can be regulated by ubiquitination or phosphorylation.^4^, ^12^ We next analysed p65 ubiquitination by immunoprecipitation of p65 followed by immunoblot analysis of ubiquitinated proteins. As shown in Figure 4E, up, left, the amount of p65‐ubiquitinated protein was clearly increased after overexpression of RND3 and disappeared when PR‐171 was used to treat cells. In parallel with the increase in p65 ubiquitination, a reduced level of p65 protein was detected in the p65 immunoprecipants in cells overexpressing RND3 compared with control cells (Figure 4E up right). In Figure 4E, down, left, the amount of p65‐ubiquitinated proteins was clearly decreased after downregulation of RND3. In parallel with the decrease in p65 ubiquitination, an increased level of p65 protein was detected in the p65 immunoprecipants in cells knocking down RND3 compared with control cells (Figure 4E down right).

Collectively, these data indicate that RND3 physically interacts with p65 and facilitates ubiquitinated‐mediated p65 protein degradation.

### 3.4 RND3 protein expression levels positively correlate with apoptosis in human GBM tissues

Abnormal activation of NF‐κB signalling is one of the most critical mechanisms of resistance against apoptosis in GBM cells.^2^ As our results showed that knocking down RND3 activated NF‐κB signalling, we next determined whether RND3 was a critical regulator of apoptosis in GBM cells.

**Figure 5:**
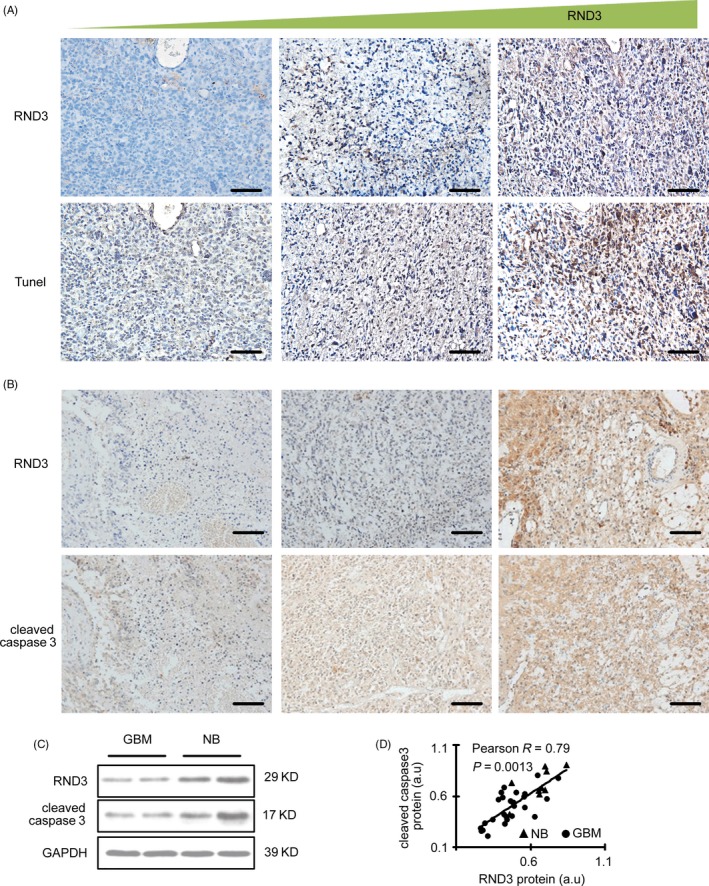
RND3 expression positively correlates with apoptosis in human GBM samples. A, RND3 expression and TUNEL staining in the same region of human GBM tissues. B, Immunostaining of RND3 and cleaved caspase 3 in the same region of human GBM tissues. C, Immunoblot analyses of RND3 and cleaved caspase 3 in human normal brain and GBM tissues. D, Correlation between RND3 and cleaved caspase 3 expression in human GBM and normal brain tissues. Scale bar = 100 µm. Triangle represents normal brain tissue (NB, n = 9). Circles represent human GBM tissue (GBM, n = 27). a.u. represents densitometric values

Caspase 3 zymogen has virtually no activity until it is cleaved by an initiator caspase after apoptotic signalling events. Cleaved caspase 3 is thus used as a marker of apoptosis.^13^ The TUNEL assay is also used to indicate apoptotic cells. To analyse the effect of RND3 on GBM cell apoptosis, the expression of RND3 and cleaved caspase 3 as well as TUNEL‐positive cells was assessed by immunohistochemical analyses in human GBM tissues. The results showed that the expression of cleaved caspase 3 and the TUNEL‐positive cell number was increased together with the increase in RND3 (Figure 5A,B), indicating that RND3 may be positively related to apoptosis of GBM cells.

To further clarify the relationship of RND3 and apoptosis, we performed immunoblotting in 27 human GBM and nine brain specimens and the correlation of RND3 and cleaved caspase 3 expression was analysed by Pearson's correlation coefficient. The result suggested a strong positive correlation between RND3 and cleaved caspase 3 protein levels (Figure 5C,D).

### 3.5 RND3 activates GBM cell apoptosis both in vitro and in vivo

**Figure 6:**
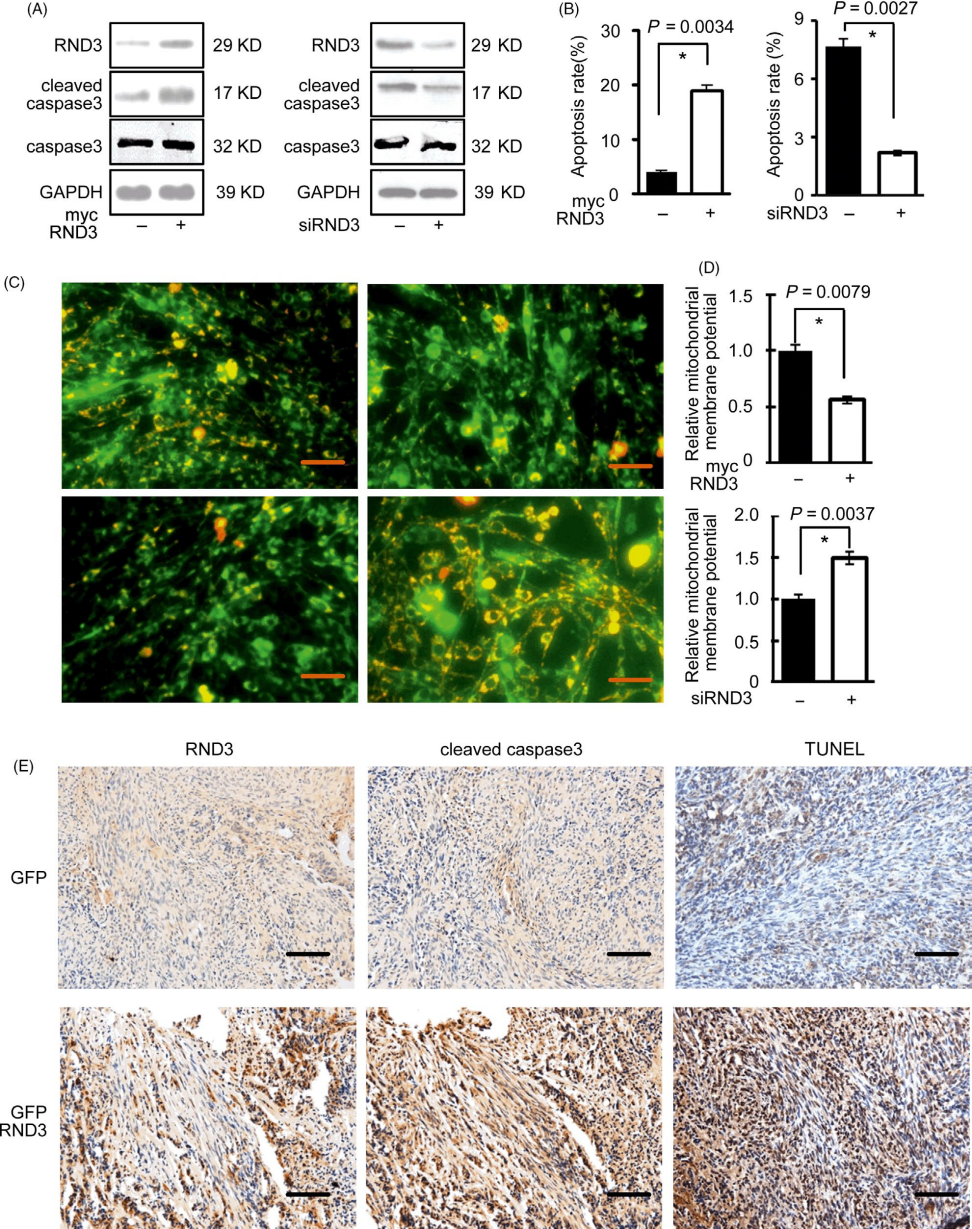
RND3 promotes GBM cell apoptosis. A, Protein expression of RND3 and cleaved caspase 3 after overexpression and knockdown of RND3 in U87 cells. B, Apoptosis rates in U87 cells after overexpression and knockdown of RND3. C, Immunofluorescence analysis of JC1, reflecting the mitochondrial membrane potential (ΔΨm), in U87 cells after overexpression and knockdown of RND3. D, Quantitative analysis of ΔΨm in U87 cells after overexpression and knockdown of RND3. E, Immunohistochemical staining of cleaved caspase 3 and TUNEL staining in nude mouse tumours from the indicated groups (n = 12 for each group). Scale bar represents 50 μm in C and 100 μm in E

The expression of cleaved caspase 3 was significantly promoted (Figure 6A) and the apoptosis rate was significantly increased when RND3 was overexpressed in U87 and U251 cells (Figures 6B and S4A,B). The opposite effects were observed with RND3 downregulation. The JC‐1 mitochondrial membrane potential assay revealed more JC‐1 aggregates in RND3 knockdown cells at 24 hours after transfection, and JC‐1 aggregates were diminished and JC‐1 monomers were increased in the cytosol of U87 cells by RND3 overexpression and vice versa (Figure 6C). Quantitative analysis of the mitochondrial membrane potential showed that the relative mitochondrial membrane potential (aggregate/monomer ratios) was decreased by RND3 overexpression and increased by RND3 downregulation (Figure 6D). These results suggest that RND3 plays a vital role in the induction of apoptosis in GBM cells.

To further support these data, the positive regulation of RND3 in apoptosis during GBM development was also examined in vivo in the human orthotopic GBM‐xenografted animal model. Significant increases in the expression level of cleaved caspase 3 and TUNEL‐positive cells were observed in GBM‐xenografted tumours overexpressing RND3 compared with the control group by immunoblot analyses (*P *< .05) (Figures 6E and S5C).

### 3.6 RND3 induces GBM cell apoptosis by inhibiting NF‐κB signalling

**Figure 7:**
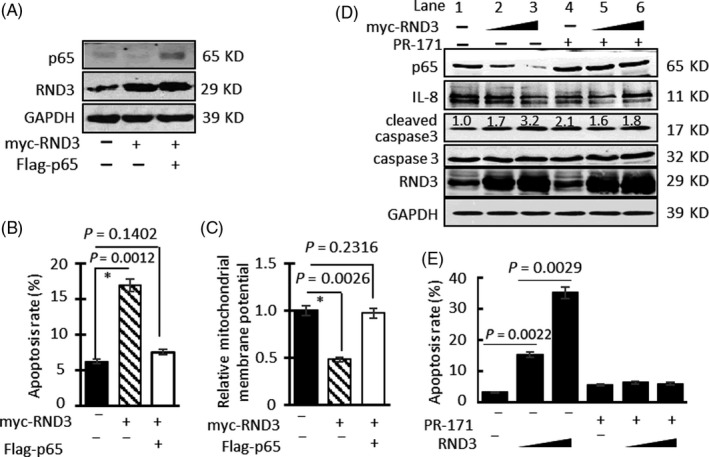
RND3 inhibits NF‐κB signalling to increase GBM cell apoptosis. A, Protein expression of RND3 and p65 after transfection of myc‐RND3 and Flag‐p65 into U87 cells. B, Apoptosis rates after transfection of myc‐RND3 and Flag‐p65 into U87 cells. C, Quantitative analysis of ΔΨm after transfection of myc‐RND3 and Flag‐p65 into U87 cells. D, Protein expression of RND3, cleaved caspase 3 and IL‐8 after overexpression of RND3 in U87 cells and treatment with or without PR‐171. E, Apoptosis rates of U87 cells after overexpression of RND3 and treatment with or without PR‐171 (20 nm)

To explore whether RND3 induced apoptosis through inhibiting NF‐κB signalling, we overexpressed RND3 in GBM cells (Figure 7A). Flow cytometry showed that the apoptosis rate was increased significantly in GBM cells overexpressing RND3 compared with controls (Figure 7B). In addition, the relative mitochondrial membrane potential was decreased in GBM cells overexpressing RND3 (Figure 7C), indicating increased apoptosis. However, when we overexpressed p65 protein by transfecting the Flag‐p65 plasmid into U87 cells, the effect of RND3 on inhibiting apoptosis and reducing the relative mitochondrial membrane potential disappeared (Figure 7B,C). Therefore, these results demonstrated that RND3 promotes apoptosis of GBM cells through inhibiting the NF‐κB signalling pathway.

We next used the PR‐17 proteasome inhibitor to block proteasome activity. Immunoblotting showed that with the increase in RND3 in U87 cells, p65 and IL‐8 protein expression decreased gradually, while cleaved caspase 3 protein expression gradually increased (Figure 7D). However, when cells were treated with PR‐171, the protein expression of p65, IL‐8 and cleaved caspase 3 did not change significantly with the increase in RND3 in U87 cells. Flow cytometry showed that, with the increase in RND3, U87 cell apoptosis rates increased gradually (Figure 7E). However, in cells treated with PR‐171, apoptosis rates did not change significantly with the increase in RND3.

Collectively, these data indicate that RND3 induces GBM cell apoptosis through downregulating p65 protein expression and inhibiting the NF‐κB signalling pathway.

## 4 DISCUSSION

RND3/Rho8/RHOE belongs to the Rnd subfamily that also includes the closely related RND1/RHO6 and RND2/RHO7/RHON proteins sharing 54%‐63% pairwise identity.^14^ Rnd proteins are atypical Rho‐GTPases compared with their typical partners in terms of protein structure and function.^15^ First, Rnd3 have N‐ and C‐terminal extensions of about 30 amino acids relative to Cdc42.^14^ Then, unlike the best‐characterized Rho family members that are post‐translationally modified by addition of a geranyl‐geranyl group (20 carbons lipid motif) at the C‐terminal CAAX (C = Cys, A = aliphatic, X = any amino acid) tetrapeptide motif, Rnd proteins are predominantly farnesylated (15 carbons lipid motif). Finally, unlike canonical members of the Rho family that are regulated by switching between an inactive GDP‐bound and an active GTP‐bound form, Rnd proteins are constitutively bound to GTP. Indeed, as these proteins lack amino acids that are critical for GTP hydrolysis.^16^

RND3 has been implicated in functions commonly regulated by Rho‐GTPases, such as remodelling of the actin cytoskeleton, and in many basic cellular processes such as cell proliferation, differentiation, survival, motility and adhesion.^17^, ^18^ RND3 also regulates cell cycle progression, which is independent of cytoskeleton remodelling, and several mechanisms may be involved, depending on the cellular context. For example, Rnd3 interacts preferentially with plexin‐B2 of the three plexin‐B proteins to induce cell rounding,^19^ whereas Rnd3 regulates Notch signalling in the ependymal epithelium, resulting in hydrocephalus through epithelial overgrowth in *RND3* null mice.^20^

We previously found that RND3 binds with the key NICD‐CSL‐MAML1 complex in Notch signalling, thereby restraining Notch signalling activity to inhibit the proliferation of GBM cells and growth of GBM.^21^ We also found that RND3 inhibits GBM cell invasion by inhibition of Snail1 signalling.^22^ In this study, we demonstrated that low expression of RND3 was one of the critical mechanisms contributing to abnormal NF‐κB signalling, which induced resistance against apoptosis in GBM cells.

A previous study showed that RND3 increased the apoptosis of U87 glioblastoma cells.^23^ RND3 overexpression induced decreases in the phosphorylation of glioblastoma protein and expression of cyclin D1, resulting in inhibition of U87 cell cycle progression.^23^ However, the authors' observations of RND3 on U87 glioblastoma cells were focused only at the cellular level. In this study, we established a human orthotopic GBM‐xenografted animal model by implantation of U251 cells with overexpression or downregulation of RND3. We found that the percentage of apoptotic cells in tumours from cells overexpressing RND3 was significantly greater than that of the control group (Figures 6E and S5C), and RND3 expression was positively correlated with apoptosis in human brain GBM samples (Figure 5). These results strongly suggest that RND3 plays an important role in GBM cell apoptosis.

**Figure 8:**
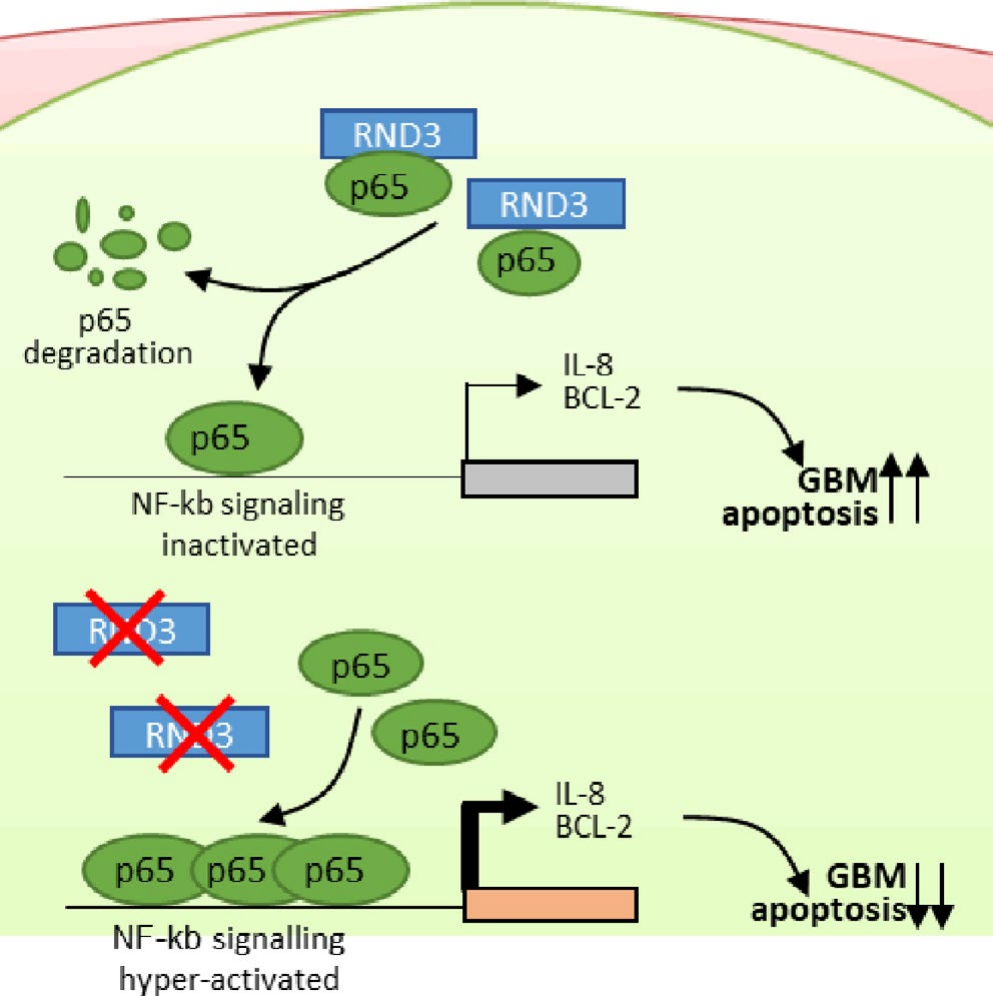
Low RND3 expression leads to activation of NF‐κB signalling and apoptosis resistance in GBM cells. High levels of RND3 in cells bind to p65, promote ubiquitination of p65 protein and inhibit NF‐κB signalling activity, whereas low expression of RND3 in cells results in reduced binding of RND3 to p65, which decreases ubiquitination of p65 protein and promotes activation of NF‐κB signalling, leading to apoptosis resistance in GBM cells

NF‐κB is one of the key signals in oncogenesis and development of human gliomas, and research is ongoing to identify selective and effective NF‐κB suppressors.^2^ The main regulatory mechanism of NF‐κB involves the IκB inhibitor that sequesters the NF‐κB complex in the cytosol. Most NF‐κB regulators exert their effects through directly or indirectly targeting IκB proteins.^4^ NF‐κB signalling is also regulated by nuclear degradation of p65^12^, ^24^ and nuclear export of the p65 protein,^25^ which are independent of IκB protein regulation. In this study, we found a key regulatory mechanism of the NF‐κB signalling pathway in GBM, namely RND3‐mediated p65 degradation. RND3 binds p65 protein and promotes the ubiquitination of p65 protein, leading to reduced p65 protein levels (Figure 8). Although no studies have yet reported the regulation of p65 by RND3 in GBM, a previous report showed that RND3 has a negative relationship with Il‑6 and Il‑8 in HepG2/ADM human hepatocellular carcinoma cells^26^ and NF‐κB activity in MCT‐induced PAH rats.^27^ Furthermore, ROCK1, a downstream effector of RND3, promotes NF‐κB activation by regulating p65 in endothelial cells.^28^, ^29^ A recent study showed that RND3 inhibits NF‐κB signalling in the mouse heart by binding to the p65‐p50 complex,^30^ although we cannot conclude that RND3 also inhibits NF‐κB signalling pathways through increasing p65 ubiquitination in GBM cells based on that study. However, these studies are consistent with our current results.

Several studies have reported different mechanisms of RND3 regulating p65. Anwar et al^28^ reported that ROCK1, a downstream effector of RND3, promoted NF‐κB activation by increasing the phosphorylation level of p65 at Ser536 in endothelial cells. However, Dai et al^30^ reported that p65 phosphorylation at Ser536 was not regulated by RND3 in heart cells and that RND3 inhibited NF‐κB activation by preventing p65 from forming a complex with other NF‐κB subunits. We found that RND3 bound to p65 and promoted the ubiquitination of p65 protein to inhibit NF‐κB signalling in GBM cells (Figures 4 and 8). To date, results from studies manipulating the expression level and effects of RND3 in various tissues and cells have been completely different.^16^, ^20^, ^31^ Therefore, the reported differences may be due to differences in the cell types.

E3 ubiquitin ligases involved in the ubiquitination of p65 in RND3 were still unknown. It has been reported that PDLIM2,^32^ copper metabolism MURR1 domain‐containing 1 (COMMD1),^24^ inhibitor of growth 4 (ING4)^33^ and makorin RING zinc finger‐2 (MKRN2),^34^ which were E3 ubiquitin ligases, had essential role in inducing p65 degradation. However, so far, only ING4 was reported to play a role in glioma tumour angiogenesis and growth. Therefore, we speculated that RND3 may promote ubiquitination of p65 protein by forming a complex with p65 and E3 ubiquitin ligase ING4.

In conclusion, we found that RND3 is a critical regulator of the NF‐κB signalling pathway in GBM. RND3 bound p65 and promoted the ubiquitination of p65 protein, leading to reduced expression of p65 protein and inhibition of the NF‐κB signalling pathway and its downstream target genes *IL‐8* and *BCL‐2* to promote apoptosis in GBM cells. In contrast, decreased levels of RND3 results in reduced binding of RND3 to p65, decreased p65 ubiquitination and increased p65 protein expression, leading to activation of the NF‐κB signalling pathway and expression of its downstream target genes *IL‐8* and *BCL‐2*, which can lead to apoptosis resistance in GBM cells (Figure 8). These findings increase our understanding of the regulation of the NF‐κB signalling pathway in GBM, thereby providing a potential new therapeutic target for GBM.

## CONFLICT OF INTEREST

None.

## AUTHOR CONTRIBUTIONS

Baohui Liu and Qianxue Chen designed the research. Qian Sun, Huimin Dong, Yuntao Li, Fan'en Yuan and sssYang Xu carried out the experimental work. Shanping Mao and Xiaoxing Xiong analysed the data and wrote the paper. All authors read and approved the final manuscript.

## Data Availability

The author confirms that this article contains a Data Availability Statement even if no data are available.
