# Supplementary material for: Small GTPase RHOE/RND3, a new critical regulator of NF‐κB signalling in glioblastoma multiforme?
Source: Cell Prolif. 2019 Jul 22;52(5):e12665. doi: 10.1111/cpr.12665 (PMC6797521; doi:10.1111/cpr.12665)
Supplement: Supplementary file 6 [file CPR-52-e12665-s006.docx]

Supplemental material
