# Supplementary material for: Small GTPase RHOE/RND3, a new critical regulator of NF‐κB signalling in glioblastoma multiforme?
Source: Cell Prolif. 2019 Jul 22;52(5):e12665. doi: 10.1111/cpr.12665 (PMC6797521; doi:10.1111/cpr.12665)
Supplement: Supplementary file 7 [file CPR-52-e12665-s007.docx]

**On line data supplement**

**Materials and Methods**

**Establishment of human GBM xenografted mouse model**

The human GBM xenografted mouse model was established according to our previous studies [^1^](#_ENREF_1)^,^ [^2^](#_ENREF_2). At 15 weeks for GFP and GFP-RND3 group, 3 weeks for shCtrl and shRND3 group after tumor implantation, nude mice were sacrificed by cervical vertebra dislocation, tumor-bearing brains were dissected, and then fixed in paraformaldehyde, dehydrated, embedded into paraffin, and cut into 5 µm-thick sections. The experimental protocol was approved by the Institutional Ethics Committee of the Faculty of Medicine at Renmin Hospital of Wuhan University and conducted in accordance with institutional guideline and regulations.

**Cell culture, transient gene transfection, and stable cell line generation**

The information of U251 and U87 cell lines, generation of U251 stable cell line, and cell culture were described in our previous study[^1^](#_ENREF_1). Cell Line Authentication by STR profiling in March 27, 2017. Proteasome inhibitor PR-171 (20nm), obtained from Selleck Chemicals (Houston, TX, USA), was used to block proteasome. The smartpool specific for siRND3 and the myc-RND3, HA-ub plasmid used in this study were described in our previous study [^1^](#_ENREF_1). The Flag-p65 plasmid was a kind gift from Dr Rong jia Zhou and Hanhua Chen.

**Immunostaining, immunoblotting, Immunofluorescence staining and co-immunoprecipitation assay**

Five microns thick sections were prepared, and double immunofluorescence staining was performed on cells according to manufacturer's instructions for the antibody. The primary antibodies used for immunostaining were as follows: anti-HA (SC-7392, SC-805, 1:200, Santa Cruz Biotechnology, Inc., CA, USA), anti-c-myc (9E10, sc-40, 1:200, Santa Cruz Biotechnology, CA, USA), anti-Flag ( F7425, 1:1000, Sigma, St. Louis, MO, USA), goat anti-RND3 (1:200, prepared in the laboratory of Professor Jiang Chang), anti-p65 (ab7970, 1:1000, Abcam Inc., Cambridge, MA, USA), anti-cleaved caspase 3 (sc-7148, 1:200, Santa Cruz Biotechnology, CA, USA), anti-BCL-2 (sc-56015, 1:200, Santa Cruz Biotechnology, CA, USA), anti-BAX (1:200, Santa Cruz Biotechnology, CA, USA), and anti-IL-8 (ab18672, , Abcam Inc., Cambridge, MA, USA). The secondary antibodies were as follows: donkey anti-goat IgG antibody conjugated with Alexa Fluor 488 (A11055, 1:1000, Invitrogen, Carlsbad, CA, USA) and goat anti-rabbit IgG antibody conjugated with Alexa Fluor 594 (A11037, 1:1000, Invitrogen, Carlsbad, CA, USA). Densitometry analysis of each protein band was performed using Band scan software, the immunohistochemical image quantifications were conducted by Leica Application Suite Imaging Software (Version 4.0, Biberach, Germany).

In Fig 4 B, Cells were co-transfected with plasmids carrying myc-RND3 (3 μg/10-cm dish) and Flag-p65 (3 μg/10-cm dish) using the Neon Transfection system (Invitrogen). At 24 h after transfection, the cells were collected and anti-FLAG M2 affinity Gel (A2220, Sigma) was used to pull down the FLAG-tagged protein. Protein A/G (sc-2003) and an anti-c-Myc antibody (9E10, sc-40) were used to pull down Myc-tagged protein. The protein expression level was detected using antibodies against c-Myc (Rabbit, sc-789, 1:200, Santa Cruz Biotechnology, CA, USA) and Flag (Rabbit, F7425, 1:1000, Sigma), according to the manufacturer's instructions.

In Fig 4 C and D, Cells were transfected with plasmids carrying myc-RND3 (3 μg/10-cm dish) or its control my plasmid (3 μg/10-cm dish) using the Neon Transfection system (Invitrogen) (in Fig 4 D, cells was also treated by PR-171 for 4h). At 24 h after transfection, cells were homogenized in RIPA buffer (50 mM Tris-HCl at pH 8.0, 0.15 M NaCl, 1 mM EDTA, and 0.5% NP-40) supplemented with protease inhibitors cocktail (Roche, 04693159001, USA). The supernatant fraction after centrifugation was immunoprecipitated with antibody P65, RND3 or preimmune IgG (Boster, AR1010, China), together with protein G PLUS-agarose (Santa Cruzs, Sc-2002). After washed with IP buffer (10 Mm Hepes,142.5 mM KCl, 5 mM MgCl, 1 mM EGTA and 0.5% NP-40), the samples were subjected to western blot analysis.

In Fig 4 E, Cells were co-transfected with plasmids carrying myc-RND3 (3 μg/10-cm dish), Flag-p65 (3 μg/10-cm dish) and HA-ub (3 μg/10-cm dish) using the Neon Transfection system (Invitrogen). At 24 h after transfection, the cells were collected and anti-FLAG M2 affinity Gel (A2220, Sigma) was used to pull down the FLAG-tagged protein. The protein expression level was detected using antibodies against HA (SC-7392, 1:200, Santa Cruz Biotechnology, CA, USA) according to the manufacturer's instructions.

**Quantitative PCR**

mRNAs were quantified by quantitative PCR analysis (Applied Biosystems StepOnePlus) using the SYBR Green PCR Master Mix containing Taq polymerase (Stratagene) as described previously (Li et al., 2010). Total RNA was prepared using TRIzol extraction kit (Gibco BRL). Forward and reverse PCR primers were (5′ to 3′) as follows: p65: forward primer: 5′-GTGGGGACTACGACCTGAATG-3′, reverse primer: 5′-GGGGCACGATTGTCAAAGATG-3′; RND3: forward primer: 5′-CTATGACCAGGGGGCAAATA-3′, reverse primer: 5′-TCTTCGCTTTGTCCTTTCGT-3′; IL-8: forward primer: 5′-ACTGAGAGTGATTGAGAGTGGAC-3′, reverse primer: 5′-CAACCCTCTGCACCCAGTTTTC-3′; BCL-2: forward：TTCTTTGAGTTCGGTGGGGTC, reverse：TGCATATTTGTTTGGGGCAGG；BAX: forward：TCCACCAAGAAGCTGAGCGAG, reverse：GTCCAGCCCATGATGGTTCT; GAPDH: forward primer: 5′-GAGTCAACGGATTTGGTCGT-3′, reverse primer: 5′-TTGATTTTGGAGGGATCTCG-3′. GAPDH expression levels were used for normalization. mRNA expression levels were determined by the 2^- ΔΔCt^ method.

**JC-1 analysis**

The JC-1 Mitochondrial Membrane Potential Assay Kit (ab113850) was purchased from Abcam. U87 cells were seeded and labeled according to the manufacturer’s protocol. Cells were then treated for 4 h with a titrated series of carbonyl cyanide 3-chlorophenylhydrazone. Both monomer and aggregate forms were detected on a Perkin Elmer-Wallac 1420 Victor 2 Multilabel plate reader. The mean and standard deviations of aggregate/monomer ratios were plotted for three replicates.

**Annexin-V-FITC assay of apoptotic cells**

The apoptosis rate of U87 and U251 cells was measured by flow cytometry. U87 and U251 cells (2 × 10^5^/well) were seeded into 6-well plates and incubated overnight. After 48 h of transfection with relevant plasmids (1 μg/well), the cells were collected and subjected to Annexin-V-FITC-propidium iodide double staining according to the manufacturer's instructions as previously described [^3^](#_ENREF_3)^,^ [^4^](#_ENREF_4). Cells were analyzed using a FACScan flow cytometer with CellQuest software (Becton-Dickinson, Mountain View, CA, USA).

Luciferase assay

A luciferase reporter vector with a promoter containing four p65-binding elements was generously provided by Jiang Chang (Texas A&M University). The luciferase assay was conducted at 36 h after transient transfection as described in our previous study [^2^](#_ENREF_2).

1 Liu, B, H Dong, X Lin, et al. RND3 promotes Snail 1 protein degradation and inhibits glioblastoma cell migration and invasion*.* Oncotarget 2016: 7(50): 82411-82423.

2 Liu, B, X Lin, X Yang, et al. Downregulation of RND3/RhoE in glioblastoma patients promotes tumorigenesis through augmentation of notch transcriptional complex activity*.* Cancer Med 2015: 4(9): 1404-16.

3 Liu, B, Z Guo, H Dong, et al. LRIG1, human EGFR inhibitor, reverses multidrug resistance through modulation of ABCB1 and ABCG2*.* Brain Res 2015: 1611: 93-100.

4 Ding, J, B Liu, Y He, et al. LRIG1 improves chemosensitivity through inhibition of BCL-2 and MnSOD in glioblastoma*.* Cell Biochem Biophys 2015: 71(1): 27-33.
